# Isolated talonavicular arthrodesis in patients with rheumatoid arthritis of the foot and tibialis posterior tendon dysfunction

**DOI:** 10.1186/1471-2474-11-38

**Published:** 2010-02-27

**Authors:** Stanislav Popelka, Rastislav Hromádka, Pavel Vavřík, Pavel Štursa, David Pokorný, David Jahoda, Antonín Sosna

**Affiliations:** 1 1st Orthopaedic Clinic, 1St Faculty of Medicine, Charles University in Prague, V Úvalu 84, 150 06, Prague 5, Czech Republic; 2 Institute of Anatomy, 1st Faculty of Medicine, Charles University in Prague, Prague, Czech Republic; 3 Department of Radiodiagnostics, Na Homolce Hospital, Prague, Czech Republic

## Abstract

**Background:**

The foot is often affected in patients with rheumatoid arthritis. Subtalar joints are involved more frequently than ankle joints. Deformities of subtalar joints often lead to painful flatfoot and valgus deformity of the heel. Major contributors to the early development of foot deformities include talonavicular joint destruction and tibialis posterior tendon dysfunction, mainly due to its rupture.

**Methods:**

Between 2002 and 2005 we performed isolated talonavicular arthrodesis in 26 patients; twenty women and six men. Tibialis posterior tendon dysfunction was diagnosed preoperatively by physical examination and by MRI. Talonavicular fusion was achieved via screws in eight patients, memory staples in twelve patients and a combination of screws and memory staples in six cases. The average duration of immobilization after the surgery was four weeks, followed by rehabilitation. Full weight bearing was allowed two to three months after surgery.

**Results:**

The mean age of the group at the time of the surgery was 43.6 years. MRI examination revealed a torn tendon in nine cases with no significant destruction of the talonavicular joint seen on X-rays. Mean of postoperative followup was 4.5 years (3 to 7 years). The mean of AOFAS Hindfoot score improved from 48.2 preoperatively to 88.6 points at the last postoperative followup. Eighteen patients had excellent results (none, mild occasional pain), six patients had moderate pain of the foot and two patients had severe pain in evaluation with the score. Complications included superficial wound infections in two patients and a nonunion developed in one case.

**Conclusions:**

Early isolated talonavicular arthrodesis provides excellent pain relief and prevents further progression of the foot deformities in patients with rheumatoid arthritis and tibialis posterior tendon dysfunction.

## Background

**Figure 1:**
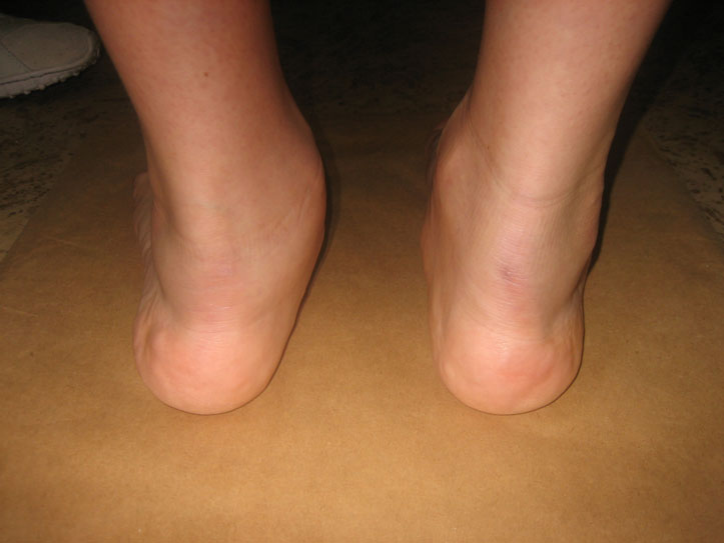
**Photographs during clinical examination**. Posterior view of the feet with valgus deviation of left heel and torn tendon of tibialis posterior muscle.

**Figure 2:**
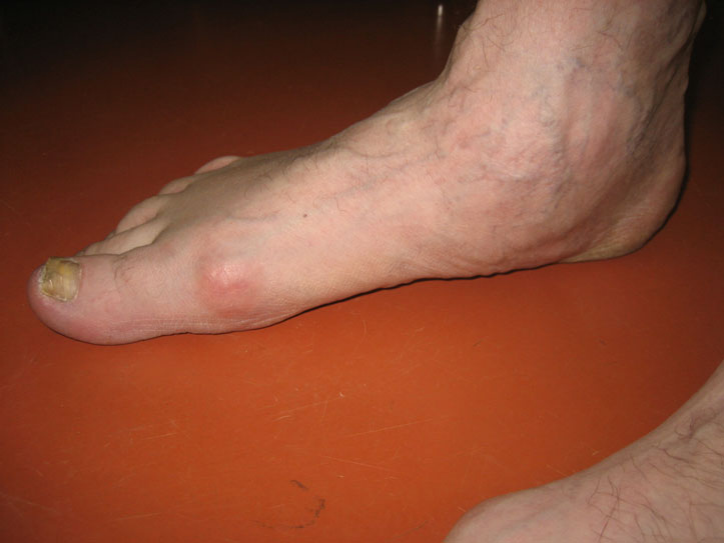
**Photographs during clinical examination**. Photograph shows the medial view of the right flatfoot in case of torn tendon of tibialis posterior muscle.

**Figure 3:**
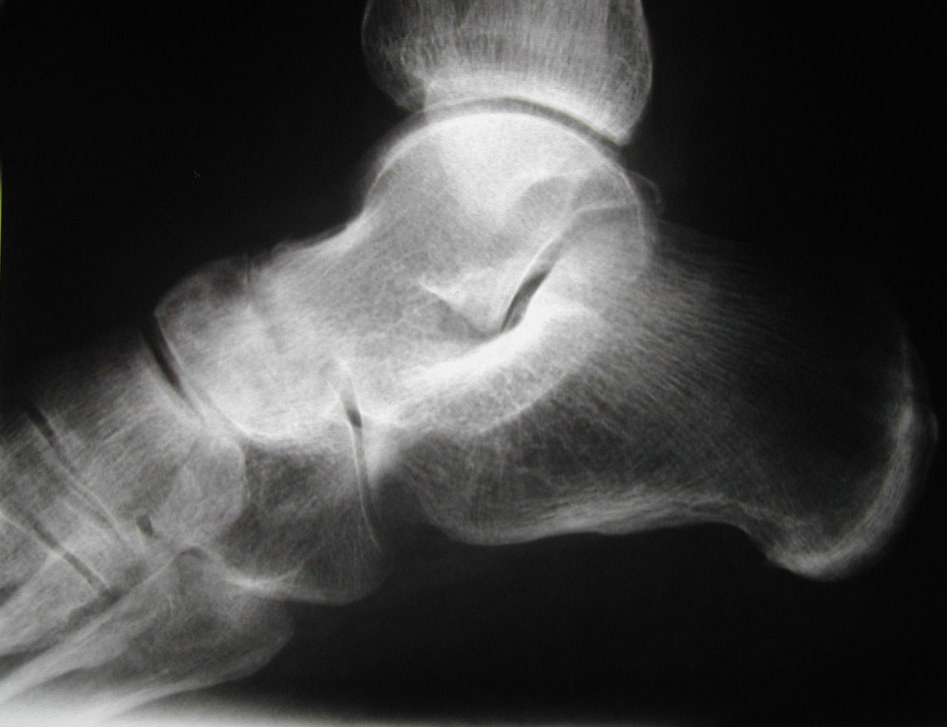
**Lateral X-ray of a foot**. Lateral X-ray of the foot shows destruction of the talonavicular joint with narrowing of the joint space.

The foot is often affected in patients with rheumatoid arthritis. Subtalar joints are involved more frequently than ankle joint [1]. Deformities of subtalar joints lead to painful flatfoot and valgus deformity of the heel. The forefoot turns in valgus deformity and the naviculare bone turns medially and plantarly (Figure 1, 2). Involvement of the talonavicular joint appears to be one of the earliest of the hindfoot joints to demonstrate deformity in rheumatoid arthritis [2,3] (Figure 3).

Major contributors to the early development of foot deformities include talonavicular joint destruction and tibialis posterior tendon dysfunction [1,3-5]. The tendon can be affected by a rheumatic process that may lead to its partial or complete rupture [6,7]. In this study we provide results after talonavicular arthrodesis in patients with rheumatoid arthritis and dysfunction of the tibialis posterior tendon.

## Methods

Between 2002 and 2005 we performed isolated talonavicular arthrodesis in 26 patients with rheumatoid arthritis (twenty women and six men) at the Orthopaedic Clinic of the 1^st ^Faculty of Medicine Charles University in Prague, Motol Teaching Hospital, Czech Republic. The study was permitted by an appropriate Ethics Committee for Multi-Centric Clinical Trials of the Motol Teaching Hospital (reference number: EK464/09). All patients in the study had planovalgus deformity and complained of pain on the dorsomedial aspect of the inner margin of the foot during weight bearing.

**Figure 4:**
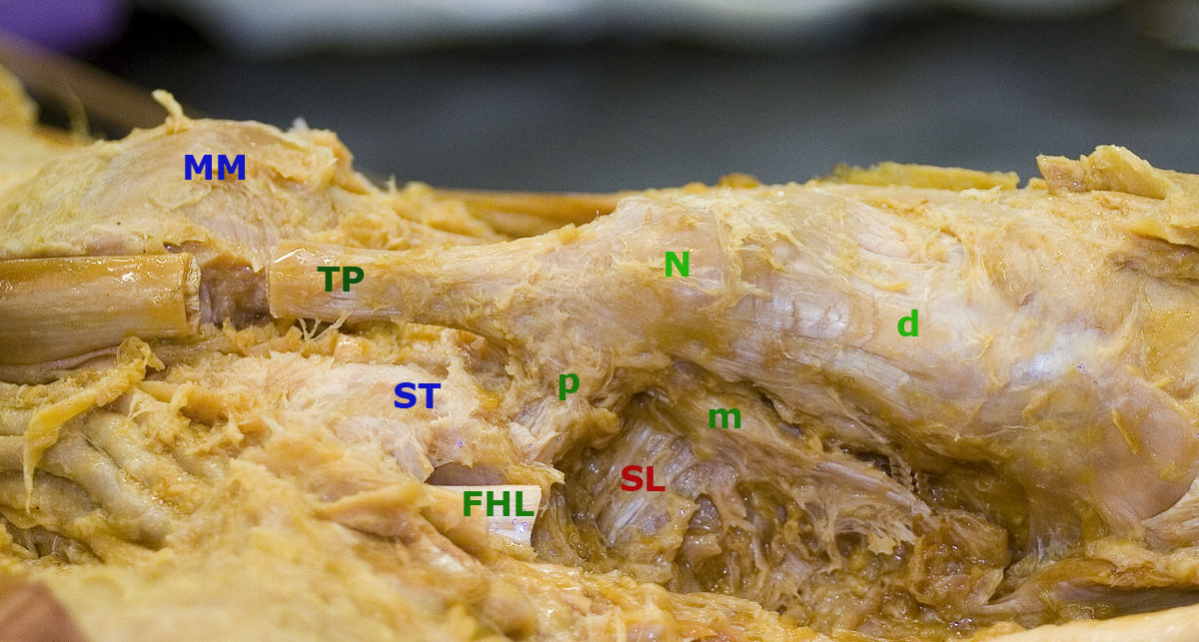
**Photographs of dissected cadaver of left foot**. Photograph of the left foot from the inferomedial aspect; Medial maleolus (MM), Tibialis posterior tendon (TP), Navicular tuberosity (N), Sustentaculum tali (ST), Flexor hallucis tendon (FHL), Spring ligament (SL); Attachment of the tendon with posterior slip (p), which is attached to the anterior margin of the sustentaculum tali, medial slip (m) to the midtarsus and distal slip (d) to the first cuneiform bone.

**Figure 5:**
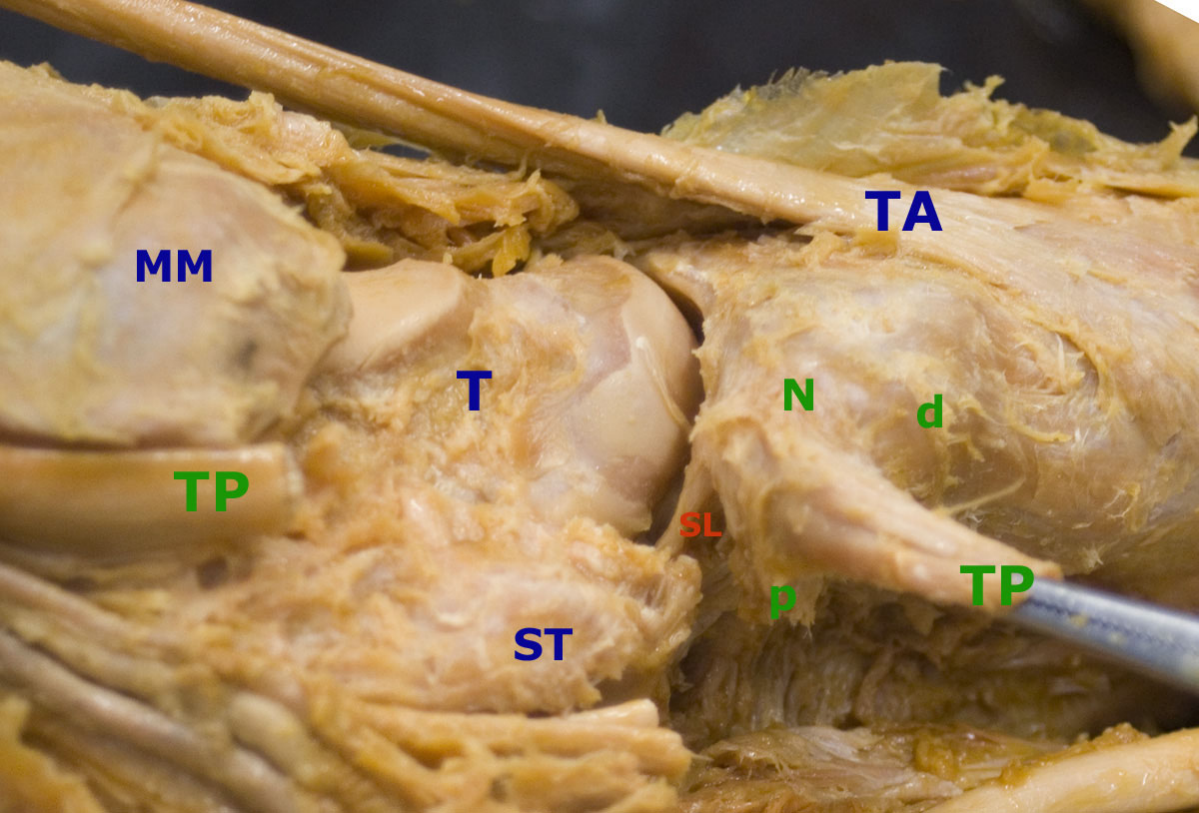
**Photographs of dissected cadaver of left foot with opened talonavicular joint**. Photograph of the left foot from the inferomedial aspect; Medial maleolus (MM), Tibialis posterior tendon (TP), Navicular tuberosity (N), Sustentaculum tali (ST), Spring ligament (SL); Attachment of the tendon with posterior slip (p), which is attached to the anterior margin of the sustentaculum tali and distal slip (d) to the first cuneiform bone.

We studied the course, attachment of the tibialis posterior tendon and its relation to the talonavicular joint on twenty cadaver specimens at the Institute of Anatomy, Charles University in Prague. Its insertion is divided into three distinct components (distal, medial and proximal). The main insertion on the navicular tuberosity goes distally to the medial cuneiform bone, the medial part into the cuneiforms, cuboid bone and the medial three metatarsal bases. The posterior part goes backwards to the sustenaculum tali of the calcaneus bone and the plantar calcaneonavicular ligament [8-11] (Figure 4, 5).

**Figure 6:**
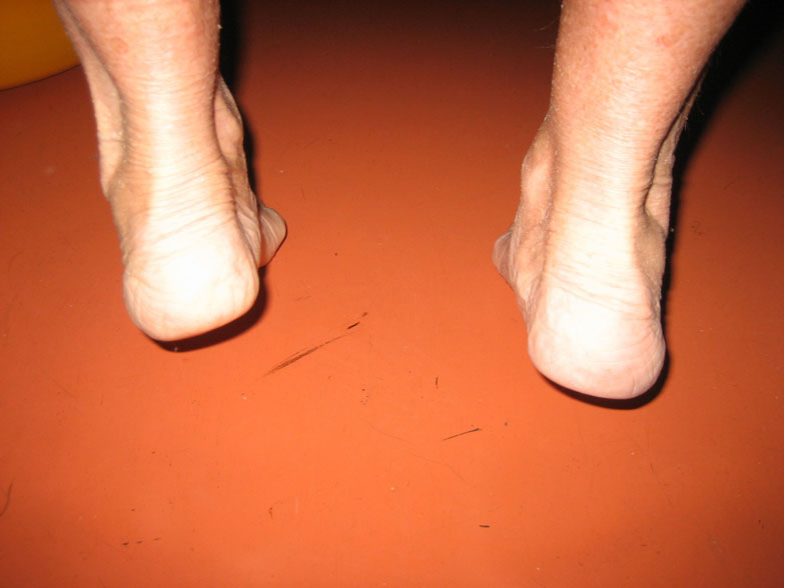
**Photograph shows clinical examination by heel rise test**. Photograph shows dysfunction of the tibialis posterior tendon during a heel-rise test. The right heel stays in the valgus position on tiptoe position.

In clinical part of the study the examination revealed signs of dysfunction of the tendon, the valgus position of the heel and the prominence of the medial foot edge. An important test is the heel rise test [12,13]. The heel stayed in the valgus position when the patient stood on his tiptoes, revealing no correction into a physiological varus position (Figure 6).

**Figure 7:**
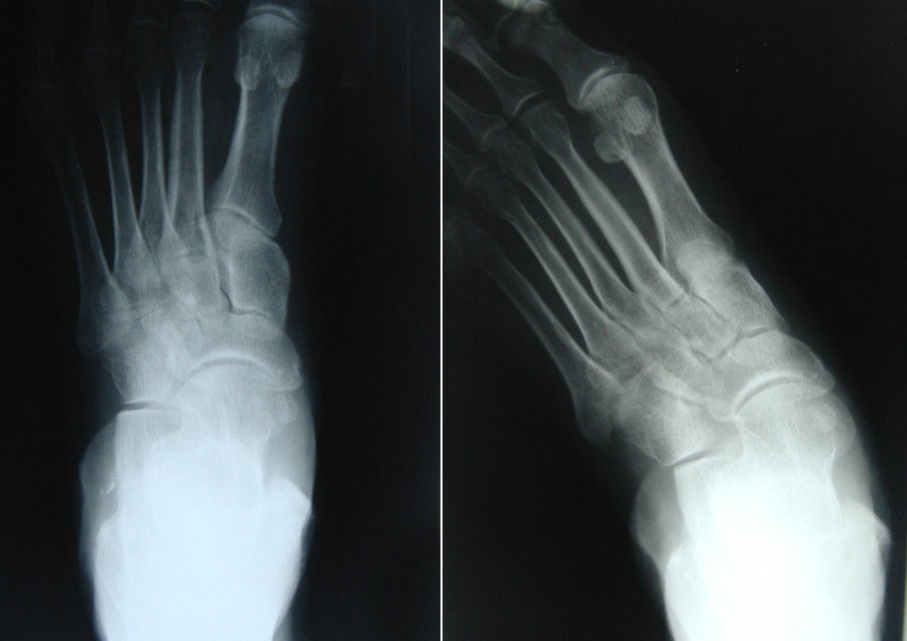
**Anteroposterior X-ray of left foot**. Right X-ray shows the standing anteroposterior view of the left foot with instability and subluxation of the talonavicular and the calcaneocuboid joints and on the left X-ray without weight bearing.

**Figure 8:**
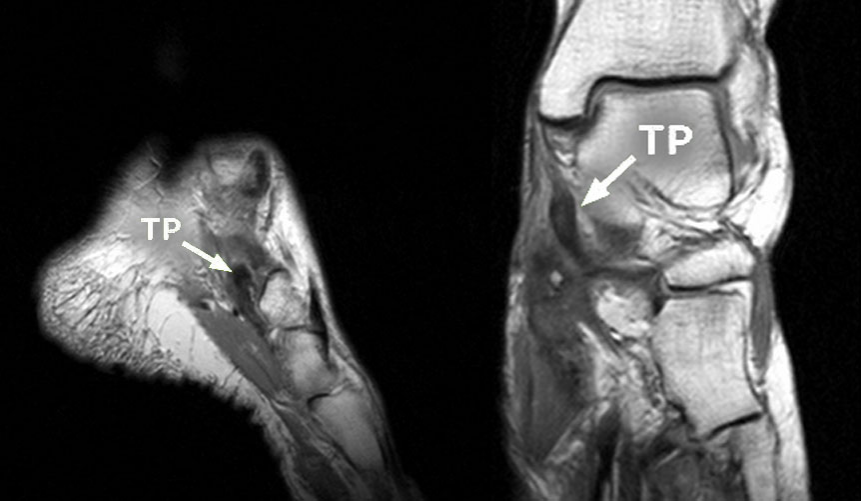
**MR examination of the left foot with torn tibialis posterior muscle tendon**. Left picture shows the frontal section of the left foot and on the right a sagittal section of it. The torn tendon of the tibialis posterior muscle (TP) is highlighted by the arrows.

All patients include to the study underwent X-ray and MRI examination. Standing AP and oblique radiographs were evaluated, showing instability of the forefoot, where the forefoot turned laterally under weight bearing conditions. These positions showed instability of the forefoot, where the forefoot turned laterally under weight bearing conditions (Figure 7). The standard MRI examination of the foot focused than on the tibialis posterior tendon prior to surgery (Figure 8).

All patients underwent physiotherapy and used individual insoles prior the surgery to release the pain of dorsomedial margin of the foot. Only patients with rheumatic destruction of the talonavicular joint and present of the pain were operated from the medial longitudinal approach at the level of the talonavicular joint. After opening the capsule of the joint we removed the remainder of cartilage from the head of the talus and naviculare bone with a shaver and chisel. In every case we revised the tibialis posterior tendon. In case of an intact tendon and the presence of synovitis, a synovectomy of the sheath was performed. When a tendon tear was found it was not possible to conduct a suture of the tendon due to the significant shortening and destruction of the remainder of the tendon. After tendon revision we reduced the space between the bones and fixed with various types of osteosynthesis (two screws, two memory staples or combination of screw and memory staple).

Following surgery a splint was applied for four to six weeks. Physical therapy followed removal of the splint (soft tissue techniques, exercises, hydrotherapy). Initially ambulation was with partial weight progressing to full weight bearing two to three months post-surgery. Patients were followed clinically and radiographically at regular intervals (one-week, six-week, three-month, six-month, one-year, two-years, etc.) after the procedure. Using standard radiographs (AP and oblique view) we evaluated the healing process of arthrodesis and the position of the ostheosynthesis. The arthrodesis had to be fused on three-month followup in both projections. We focused not only on the talonavicular joint, but additionally on whether the arthritis had spread to other foot joints and whether the deformity of the foot had progressed. We used AOFAS Hindfoot score [14] for evaluation subjective and objective findings.

## Results

We assessed the patients in 2008, after a longer time period had elapsed since their operations. The average age of patients at the time of the surgery was 43.6 years. The right foot was operated on in fifteen cases and the left foot in eleven.

Signs of arthritis of the talonavicular joint were visible on the X-rays in 13 cases, while in the other 13 cases the talonavicular joint showed minimal destruction prior to surgery. We found torn tibialis posterior tendons in nine patients on MRI.

During the surgery, we found complete ruptures of the tibialis posterior tendon in nine cases, and in seven cases the tendon was significantly weakened and affected by the rheumatic process. The tendon showed no signs of impairment in the remaining ten cases. The rupture was always close to the tendon insertion on the naviculare bone as was previously published [15]. Two screws in eight cases; two memory staples (DePuy) in twelve cases and a combination of screw and memory staples in six cases (DePuy) were used to stabilize the arthrodesis.

Mean followup was 4.5 years (range, 3 to 7 years). AOFAS Hindfoot score [14] improved from 48.2 points preoperatively to 88.6 points at the last postoperative followup. Eighteen patients had excellent results (none, mild occasional pain), six patients had moderate pain of the foot and two patients had severe pain. We found union of the arthrodesis on radiographs in all cases except one case at six-month followup. The nonunion developed in the case, when the arthrodesis was fixed by two screws.

In two cases other joints, namely the talocalcaneal and calcaneocuboid joints, were affected at three and six years after surgery, respectively. Complications included superficial wound infections in two patients, treated successfully with oral antibiotics.

## Discussion

The foot is often affected in patients with rheumatoid arthritis, as stated by many authors [2,4,16-18]. Sometimes, this can be the first sign of the disease. The early symptoms include pain and gradual development of forefoot deformity.

In patients with rheumatoid arthritis the talonavicular joint is often the first joint affected. Many authors [2-5,19,20] published good results with isolated talonavicular arthrodesis in patients with rheumatoid arthritis. They quote excellent results in 95% of the cases when patients had no subjective problems and the foot was in a satisfactory position. Overall subjective satisfaction of patients in our study was 92.3%, which correlated with prior studies [2,4] and only two patients reported severe foot pain (7.7%).

**Figure 9:**
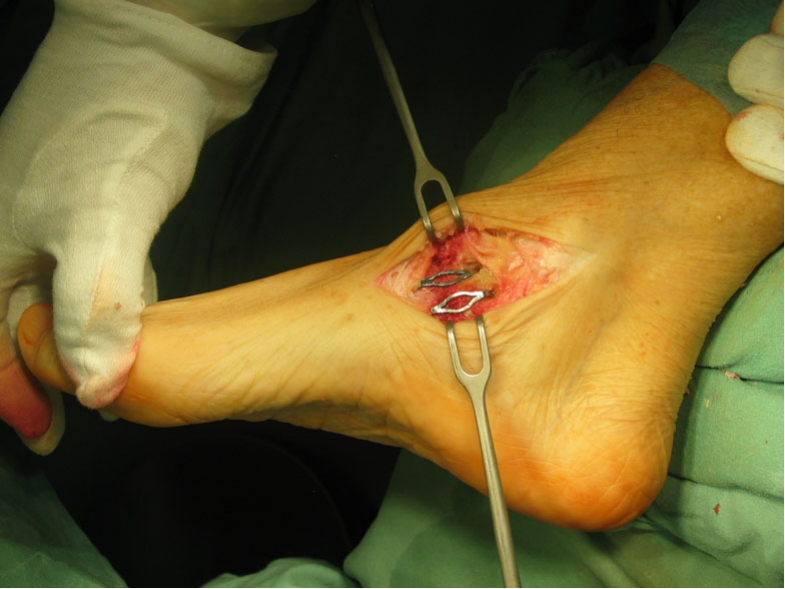
**The fixation of arthrodesis by two memory staples**. Photograph shows the fixation of the arthrodesis during the surgery by two memory staples (DePuy).

**Figure 10:**
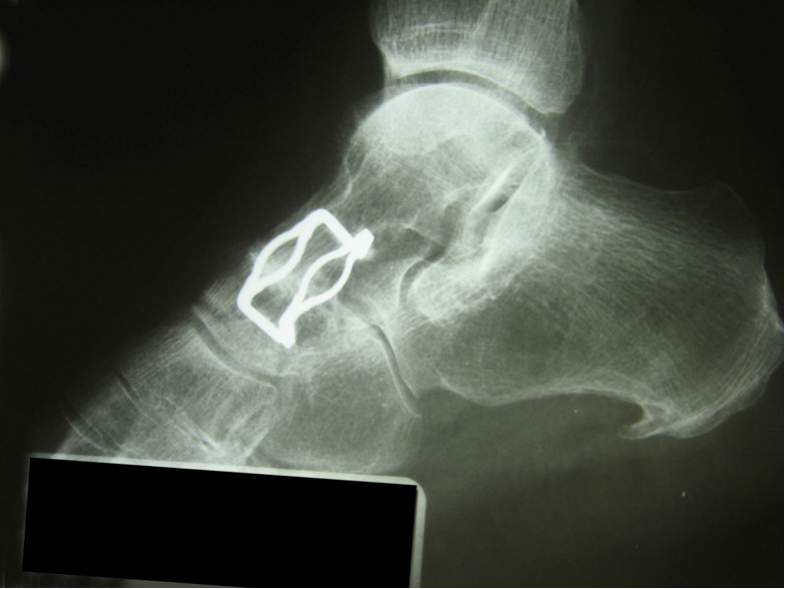
**The fixation of arthrodesis by two memory staples on X-ray**. Radiograph shows the healed arthrodesis three years after the procedure.

Various osteosynthetic materials are used for fixation of the arthrodesis. The authors usually used two screws while we used two screws or a combination of one screw with one memory staple in the beginning. Later, we stabilize the arthrodesis with two memory staples. We believe that two memory staples provide excellent stability of the fixation and quick healing process (Figure 9, 10). We found clear union of the arthrodesis on three-month followup in these cases without developing a radiolucent line or a sclerosis of surrounding bones. Some authors use corticospongious iliac bone graft and a screw or plate fixation [3,16,21,22]. Bone grafts were not used in our group of patients.

We found a complete rupture of the tibialis posterior tendon in nine patients on MRI and during the operation. During clinical examination of these patients, signs of dysfunction of the tibialis posterior tendon were present [8,9,13,18,23,24]. In all these cases, the talonavicular joint capsule was significantly disengaged.

Elboar et al. [16] also assessed the effects of this isolated arthrodesis on the development of changes in other joints of the foot. They did not find any relation between talonavicular joint arthrodesis and progression of changes in the other joints of the foot. In our set of patients we recorded the progression of deformities and changes in surrounding joints in two patients (three and six years after the operation). Subtalar joint arthrodesis is planned in both cases.

Early isolated talonavicular arthrodesis prevents further deviations of the forefoot and deformities of other joints. It is necessary to perform it in the early stages, while the deformity is flexible and the foot can be returned to the neutral position. In case of a fixed deformity, a triple-desis of the subtalar joint is necessary [1]. Using cadaver specimens, Suckel et al. [25,26] measured pressure distribution in the ankle joint after triple-desis and talonavicular arthrodesis. After triple-desis, pressure in the ankle joint increases. This may lead to the development of its degenerative changes and further destruction of all joints of the foot.

According to the literature, pseudoarthrosis occurs in 3-5% [2,4]. Development of pseudoarthrosis is more frequent in the case of talonavicular arthrodesis than in the case of talocalcaneal arthrodesis. In talocalcaneal arthrodesis the removed surface is larger and it is perpendicular to the load direction. On the contrary, in the talonavicular joint the articulation is different - the contact surface is much small with less favorable shearing forces present. In our set of patients we recorded only one case of pseudoarthrosis (3.8%).

## Conclusions

Isolated talonavicular arthrodesis is an effective method of treatment of talonavicular arthritis regarding pain relief and functional improvement. Early fusion of the talonavicular joint appears to prevent further progression of foot deformities. In nine patients in our cohort we encountered rupture of the tibialis posterior muscle tendon. This decreases the biomechanical stability of the talonavicular joint while increasing the shear load of the joint and destabilizing the Chopart's joint.

## Competing interests

The authors declare that they have no competing interests.

## Authors' contributions

The final manuscript has been read and approved by all authors. SP - main author; prepared and wrote down the manuscript. RH - anatomical study of tibialis posterior muscle; prepared and dissected cadaver specimens. PV - anatomical study of tibialis posterior muscle; orthopedics part of dissections. PŠ - examinations radiographs and MRI pictures; DP - surgeon who operated patients in the study. DJ - surgeon who operated patients in the study. AS - head of the clinic; co-author of manuscript.

## Consent of patient

Written consent was obtained from the patient or their relative for publication of the patient's details.

## Pre-publication history

The pre-publication history for this paper can be accessed here:

http://www.biomedcentral.com/1471-2474/11/38/prepub
